# Therapeutic Use and Molecular Aspects of Ivabradine in Cardiac Remodeling: A Review

**DOI:** 10.3390/ijms24032801

**Published:** 2023-02-01

**Authors:** Yusof Kamisah, Hamat H. Che Hassan

**Affiliations:** 1 Department of Pharmacology, Faculty of Medicine, Universiti Kebangsaan Malaysia, Kuala Lumpur 56000, Malaysia; 2 Department of Medicine, Faculty of Medicine, Universiti Kebangsaan Malaysia, Kuala Lumpur 56000, Malaysia

**Keywords:** heart failure, left ventricular dysfunction, myocardial fibrosis, cardiac function, cardiac structure

## Abstract

Cardiac remodeling can cause ventricular dysfunction and progress to heart failure, a cardiovascular disease that claims many lives globally. Ivabradine, a funny channel (I*_f_*) inhibitor, is used in patients with chronic heart failure as an adjunct to other heart failure medications. This review aims to gather updated information regarding the therapeutic use and mechanism of action of ivabradine in heart failure. The drug reduces elevated resting heart rate, which is linked to increased morbidity and mortality in patients with heart failure. Its use is associated with improved cardiac function, structure, and quality of life in the patients. Ivabradine exerts several pleiotropic effects, including an antiremodeling property, which are independent of its principal heart-rate-reducing effects. Its suppressive effects on cardiac remodeling have been demonstrated in animal models of cardiac remodeling and heart failure. It reduces myocardial fibrosis, apoptosis, inflammation, and oxidative stress as well as increases autophagy in the animals. It also modulates myocardial calcium homeostasis, neurohumoral systems, and energy metabolism. However, its role in improving heart failure remains unclear. Therefore, elucidating its molecular mechanisms is imperative and would aid in the design of future studies.

## 1. Introduction

Heart failure is the leading cause of death worldwide. It is the costliest disease and has become a socioeconomic burden globally [1]. Its prevalence is estimated to be approximately 1–2% in developed countries [2], claiming nearly nine million lives in 2019 [3]. It causes repeated hospitalization [4]; it commonly arises from complications of other ailments, such as ischemic heart disease and uncontrolled hypertension [5]. 

**Figure 1:**
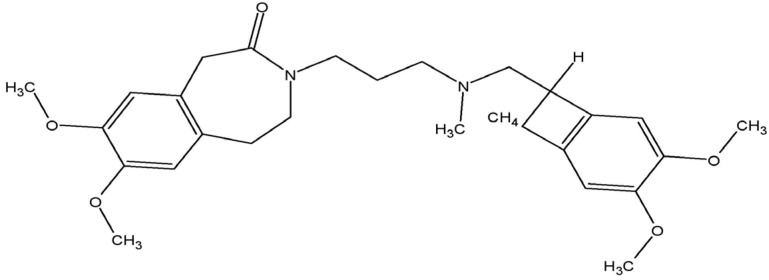
Molecular structure of ivabradine.

A high resting heart rate increases the risk of adverse outcomes (morbidity and mortality) in patients with heart failure [6]. Thus, besides the reduction in excessive neurohumoral activation in patients with heart failure, slowing down the heart rate seems to be another therapeutic option [7,8]. This target is commonly achieved using β-blockers. However, clinically, uptitration of the drugs to the optimal dosage is complicated due to side effects [9]. Ivabradine (Figure 1), marketed as Procoralan^®^, Ivabid^®^, or Ivazine^®^, is a pure heart rate reducer [7]. The drug was originally approved for the treatment of angina pectoris; however, since 2005, it has been used as an adjunct therapy in patients with stable symptomatic heart failure with reduced ejection fraction (HFrEF) with concomitant high resting heart rate (>70 beats per min), which is an independent predictor for cardiovascular disease [7,9]. 

Cardiac remodeling is a process that involves structural changes affecting the size and shape of the myocardium, characterized by cardiac hypertrophy. Cellular and molecular changes can lead to cardiac dysfunction [10]. Animal studies demonstrated that ivabradine therapy reduced these changes, evidenced by a reduction in growth factors, collagen, and matrix metalloproteinase (MMP) expression, the increase in which leads to myocardial fibrosis in animal models of heart failure [11,12]. It also ameliorated myocardial inflammation, apoptosis, and oxidative stress as well as improved myocardial biogenesis in the remodeled hearts [12,13,14,15], all factors potentially contributing to the antiremodeling effects. However, the molecular and cellular effects of ivabradine on cardiac remodeling have not been investigated in depth and remain unclear. This review aims to outline the updates on the therapeutic use of ivabradine and its possible mechanistic properties in cardiac remodeling and heart failure. Comprehending the molecular mechanism of ivabradine could promote future research and help to strategize its clinical use.

## 2. Clinical Outcomes of Ivabradine Therapy

Increased mortality due to cardiovascular events and frequent hospitalization are common in patients with heart failure. In addition, the progression of heart failure reduces the quality of life of these patients. Many clinical trials, such as the Systolic Heart Failure Treatment with the I*_f_* Inhibitor Ivabradine Trial (SHIFT), Long-term Treatment with Ivabradine in Ambulatory Patients with Chronic Heart Failure (RELIf-CHF), Study Assessing the Morbidity-Mortality Benefits of the I_f_ Inhibitor Ivabradine in Patients with Coronary Artery Disease (SIGNIFY), and Morbidity-mortality Evaluation of the I_f_ Inhibitor Ivabradine in Patients with Coronary Disease and Left Ventricular Dysfunction (BEAUTIFUL), have been conducted to assess the outcomes. Heart failure patients taking ivabradine have a reduced risk, frequency, and length of hospitalization due to worsening heart failure, other cardiovascular disease, or other co-morbidities, compared with those who do not take ivabradine (Table 1) [16,17,18,19,20]. 

However, the effects of ivabradine on mortality rate in these patients were inconsistent. Most studies including principal trials (SHIFT, BEAUTIFUL, and SIGNIFY) reported that ivabradine therapy failed to decrease the rate of death due to cardiovascular disease or other causes despite the reduction in hospitalization [16,22,24,25,30,37]. Nevertheless, three studies reported positive outcomes on mortality due to cardiovascular events or heart failure following ivabradine treatment [19,21,28] in patients with a resting heart rate higher than 75 beats per minute; however, these outcomes were not observed in patients with a lower resting heart rate [21]. An elevated resting heart rate predisposes a patient to developing left ventricular systolic dysfunction [38]. Therefore, a reduction in heart rate by ivabradine would potentiate systolic function, leading to a reduction in the severity of the disease, evidenced by a lower New York Heart Association class [18,19,20,23,28,29,32,33,34,36]. Furthermore, this would decrease hospital readmissions due to the worsening of heart failure.

In terms of quality of life, ivabradine therapy improved global assessment, either by patient self-assessment or assessment by their physician (Table 1) [19]. This translated to increased health-related quality of life evidenced by a reduction in heart-failure-associated symptoms and improvements in physical, social, and emotional functioning, well-being, vitality, and general health. Furthermore, these improvements led to increased mental health scores [20,31,32,34]. A clinical trial was conducted on children (aged 6 months to 18 years old) with dilated cardiomyopathy. It was reported that ivabradine improved the quality of life in these children [36]. In summary, ivabradine therapy improves heart-failure-associated symptoms, resulting in a better quality of life for patients, but with limited success in reducing mortality in these patients.

## 3. Effects on Cardiac Function

As previously mentioned, one of the primary targets in patients with chronic heart failure is a reduction in excessive neurohumoral activation, particularly in terms of the attenuation of the sympathetic system and renin–angiotensin–aldosterone system activation. The use of β-blockers not only decreases the heart rate but also decreases cardiac contractility and blood pressure in these patients. In addition, high doses of β-blockers result in reduced patient tolerance for the drug’s side effects, which include fatigue and hypotension [9]. Ivabradine is used as a second-line treatment in addition to β-blockers and other drugs used for heart failure treatment [9,39]. The heart-rate-lowering property of ivabradine at doses of 5–7.5 mg twice daily has been observed in many clinical studies in both acute and chronic heart failure patients (Table 2) [18,28,40,41]. However, the effect was not apparent in heart failure patients with a resting heart rate lower than 75 beats per minute [21], suggesting that it has the potential to not cause bradycardia.

In contrast with β-blockers, ivabradine does not affect blood pressure [18,28,33,44] or myocardial contractility [9] in patients with heart failure. The reduction in heart rate observed in the patients taking ivabradine leads to a decrease in left ventricular end-diastolic volume (LVEDV) [19,46] and the ratio of early diastolic mitral inflow velocity to early diastolic mitral annular velocity (E/E′) (Table 2) [44]. However, other parameters of diastolic function, such as ratios of early-to-late diastolic mitral inflow velocity (E/A) and early diastolic mitral inflow velocity to early diastolic velocity of the septal mitral annulus (E/e′), were not significantly altered by ivabradine [27,44]. Following the improvement in diastolic function, ivabradine indirectly ameliorates systolic work in patients, manifested by increased left ventricular ejection fraction (LVEF), end-systolic elastance (Ees), and stroke volume and decreased end-systolic volume (LVESV) [18,19,23,35,40,41,46]. However, several studies demonstrated unaltered Ees [27,45] and LEVF [28,43,44] following ivabradine therapy. 

Studies exploring the impact of ivabradine on right ventricular function in patients with heart failure are lacking. Only Gul et al. [47] reported that the drug therapy ameliorated right ventricular function based on the improvement of strain rate and global longitudinal strain parameters. However, it was a small non-randomized study involving only two centers. The improvement in the right ventricular function could arise from the improvement of the left ventricular performance, which decreases the right ventricular afterload. 

Left ventricular dysfunction is closely related to prolonged atrial conduction time, with the latter increasing the risk of atrial fibrillation in patients with heart failure [48]. Only one study investigated the effects of ivabradine on atrial mechanical function. The delay in interatrial and right intra-atrial conduction was significantly reduced in patients with systolic heart failure after 3 months on ivabradine [44]. Furthermore, the drug improved atrial electromechanical function in these patients, indicated by decreased left atrial active emptying volume and fraction and decreased duration of onset of the P wave to the beginning of the late diastolic wave at the septal and lateral mitral annulus and right ventricular tricuspid annulus [44]. These observations suggest that ivabradine may exert beneficial effects on myocardial atrial performance, with the potential to reduce the risk of developing arrhythmia in patients with heart failure. However, a recent meta-analysis that included 13 clinical trials inferred that regardless of the dose, ivabradine increased the incidence of atrial fibrillation in patients. However, the drug is effective in preventing post-operative atrial fibrillation [49]. Nonetheless, more clinical studies should be conducted to confirm these findings. Collectively, the findings obtained to date suggest that ivabradine may restore left ventricular, right ventricular, and left atrial function in failing hearts.

The cardioprotective effects of ivabradine were also demonstrated in animal studies. Ivabradine administered at 10 mg/kg/day in drinking water for 2–12 weeks produced improvements in cardiac function in various animal models of cardiac remodeling (Table 3).

It enhanced systolic function by increasing stroke volume, LVEF, left ventricular fractional shortening (LVFS), systolic pressure (LVSP) and developed pressure (LVDP), maximal rate of fall (−dp/dt_max_) and rise (+dp/dt_max_) of left ventricular pressure, and LVESV in these animal models [11,12,13,51,53,57,59,60,62,67].

Left ventricular dysfunction, commonly seen in heart failure, is characterized by impaired left ventricular filling capacity [75]. Ivabradine potentiates diastolic work by increasing the diastolic filling time [52,64] and decreasing left ventricular diastolic wall stress [61] in chronic-hypertension-induced cardiac hypertrophy and myocardial-infarction-induced cardiac remodeling in animals. Reductions in left ventricular end-diastolic pressure (LVEDP), isovolumetric relaxation time (IVRT), Tau (early relaxation), LVEDV, and E/E′ were also noted (Table 3) [11,51,52,56,58,61,63,65,66,67].

The potential benefits of ivabradine were further investigated in right ventricular dysfunction. In a pulmonary-hypertension-induced heart failure rat model, oral administration of 10 mg/kg/day ivabradine for 3 weeks improved right ventricular systolic function evidenced by reduced maximum tricuspid systolic annular excursion (tTAPSE) and isovolumic contraction time (IVCT) and increased systolic tissue wave velocity (S’), stroke volume, and cardiac output (Table 3) [69,72]. Altered right ventricular +dp/dt_max_ and −dp/dt_max_ values were also reversed in the rats [72]. In addition, right ventricular diastolic function was preserved based on the improvement in IVRT, right ventricular end-diastolic pressure (RVEDP), and Tau [72]. Similar findings were noted in SU5416 (a tyrosine kinase inhibitor) plus hypoxia-induced cardiac remodeling and right-ventricular-pressure-overload-induced cardiac remodeling [72]. In primary right ventricular cardiomyocytes, ivabradine (0.01–1 μM) reduced beating frequency without affecting the beating amplitude [72], confirming its heart-rate-lowering effects with no direct impact on contractility.

Altered calcium uptake into the sarcoplasmic reticulum hinders contractile performance [76]. Sarcoplasmic/endoplasmic reticulum calcium ATPase 2a (SERCA2a) and phosphorylated phospholamban are two proteins that regulate calcium uptake into the sarcoplasmic reticulum [77,78]. Improved systolic work by ivabradine may partially be attributed to its influence on myocardial calcium regulation. The drug decreased the expression of SERCA2a and phosphorylated phospholamban in rats that were exposed to monocrotaline-induced pulmonary hypertension to induce cardiac remodeling [72]. The transporting function of SERCA was increased following ivabradine treatment without affecting the function of sodium–calcium exchanger (NCX) and sarcoplasmic reticulum calcium storage. The net effect was an increase in calcium transient amplitude in the heart [61]. NCX mediates the exchange of Na^+^ and Ca^2+^ when the extracellular Na^+^ is high due to the activity of Na^+^/K^+^-ATPase, which transports Na^+^ out of cells in favor of transporting K^+^ into cells [79]. Calcium is also required for ATP generation in the mitochondria. Increased mitochondrial calcium uptake enhances ATP production, leading to improvements in energy metabolism and supply to contractile proteins during systolic and diastolic actions [80]. However, studies investigating the role of ivabradine in mitochondrial calcium uptake and release are lacking. 

Based on the reported findings, it can be stipulated that ivabradine confers protection against left and right ventricular dysfunction in animal studies, which confirms the clinical observations. These findings may partially be attributable to the effects of ivabradine on myocardial calcium homeostasis. Other factors that should be investigated are the influence of the drug on other calcium regulators, such Na^+^/K^+^-ATPase, ryanodine receptor 2, which facilitates Ca^2+^ release from the sarcoplasmic reticulum [77], and Ca^2+^/calmodulin-dependent protein kinase II (Ca^2+^/CaMKII), which is involved in Ca^2+^ signal transduction [81]. Its effects on mitochondrial voltage-dependent anion channel 1, calcium uniporter, and calcium uptake proteins—mitochondrial calcium regulatory proteins [80]—should also be studied. 

## 4. Effects on the Cardiac Electrical Activity and Neurohumoral Systems

The heart-rate-lowering property of ivabradine arises from its selective inhibition of the I*_f_* current, also known as the “funny current”, in the right sinoatrial node [9,82], which is a constituent of the cardiac conduction system involved in the autogeneration of cardiac impulses [83]. The I*_f_* current involves the influx of Na^+^ and K^+^ that travel through hyperpolarization-activated cyclic nucleotide-gated (HCN) channels. There are four isoforms of HCN—HCN1, HCN2, HCN3, and HCN4 [84]—with HCN4 being highly localized in the human heart [85]. HCN4 expression is upregulated in failing human hearts [59].

Ivabradine downregulates HCN4 expression in animal models of heart failure (Table 4). Paterek et al. [59] and Gomes et al. [70] demonstrated that the decrease in heart rate was accompanied by a downregulation of left ventricular *HCN4* expression in rats induced with heart failure. However, similar findings were not observed in a study by Kakehi et al. [11]; they reported reduced expression of *HCN2* channels in the right atrium of hypertensive rats with heart failure treated with ivabradine. Both HCN2 and HCN4 are similarly expressed in rat hearts [86]. The HCN4 isoform has proarrhythmic potential [59]. Caveolin 3, a protein that is localized in cardiomyocyte caveolae, forms a complex with HCN4, leading to β-adrenergic blockade [87]. A recent study has reported that ivabradine stabilizes the formation of the caveolin–HCN4 complex [74], thereby inhibiting the I*_f_* current and leading to a reduction in heart rate. Therefore, its inhibition is advantageous to mitigate the risk of developing arrhythmia.

HCN channels and the I*_f_* current are also present in the atrioventricular nodes and Purkinje fibers [88]. Ivabradine lengthened QRS intervals in anesthetized mice [50]. It also reduced ventricular rate by prolonging atria-His and PR intervals during atrial fibrillation in animal models [89]. The findings suggest that ivabradine may affect intraventricular conduction acceleration. The particular effects of ivabradine are currently investigated in the BRAKE-AF multicenter, randomized, and controlled phase III clinical trial by a research group in Spain. The trial aims to assess the effects of the drug on chronic heart rate control in patients with uncontrolled persistent atrial fibrillation [90]. The outcomes of the trial may affirm the findings from animal studies and its therapeutic use clinically.

**Figure 2:**
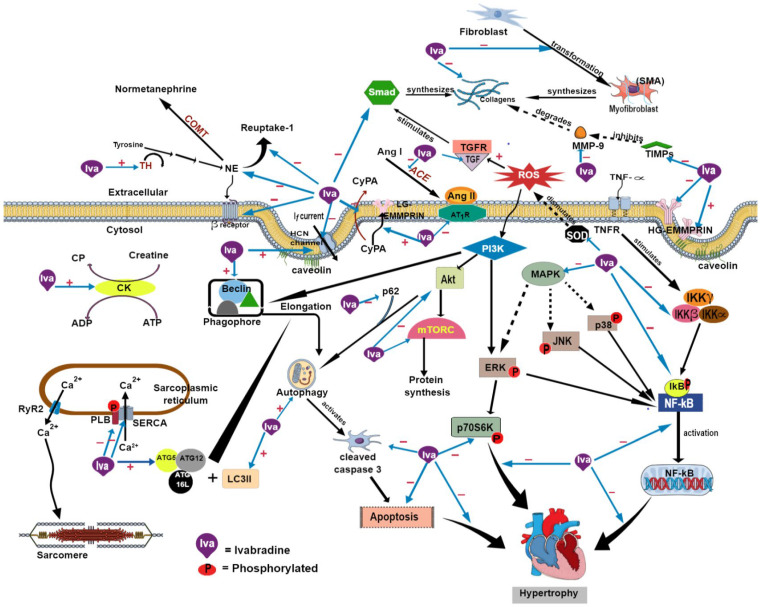
Possible molecular sites of action of ivabradine on cardiac remodeling. ACE, angiotensin-converting enzyme; ADP, adenosine diphosphate; Akt, protein kinase; Ang, angiotensin; ATG, autophagy-related; AT_1_R, angiotensin II type 1 receptor; ATP, adenosine triphosphate; COMT, catechol-O-methyltransferase; CK, creatine kinase; CP, creatine phosphate; CyPA, cyclophilin A; ERK, extracellular signal-regulated kinase; HCN, hyperpolarization-activated cyclic nucleotide-gated; IKB; nuclear factor of kappa light polypeptide gene enhancer in B-cells inhibitor; HG-EMMPRIN, high-glycosylated extracellular matrix metalloproteinase inducer; IKKγ, inhibitor of nuclear factor kappa-B kinase subunit γ; IKKβ, inhibitor of nuclear factor kappa-B kinase subunit β; IKKBα, inhibitor of nuclear factor kappa-B kinase subunit α; IκBα, nuclear factor of kappa light polypeptide gene enhancer in B-cells inhibitor α; t-IκBα, total inhibitor of nuclear factor kappa-B kinase subunit α/β and IκBα; nuclear factor of kappa light polypeptide gene enhancer in B-cells inhibitor α; p-IKKα/β, inhibitor of nuclear factor kappa-B kinase subunit α/β; JNK, c-Jun N-terminal kinase; LG-EMMPRIN, low-glycosylated extracellular matrix metalloproteinase inducer; LC3II, microtubule-associated protein light chain 3 II; MAPK, mitogen-activated protein kinase; MMP, matrix metalloproteinase; mTORC, mammalian target of rapamycin complex; NE, norepinephrine; NF-κB, nuclear factor-kappa B; PLB, phospholambam; ROS, reactive oxygen species; p-70S6K, phosphorylated protein S6 kinase beta-1; PI3K, phosphatidylinositol 3-kinase; RyR2, ryanodine receptor 2; SERCA2a, Sarcoplasmic/Endoplasmic reticulum calcium ATPase 2a; SMA, α-smooth muscle actin; Smad, the small mothers against decapentaplegic; SOD, superoxide dismutase; TGF, transforming growth factor; TGFR, transforming growth factor receptor; TH, tyrosine hydroxylase; TIMPs, tissue inhibitor of metalloproteinase; TNF-α, tumor necrosis factor α; TNFR, tumor necrosis factor receptor; −, inhibits; +, promotes/increase. Figure created in the Mind the Graph Platform, available at www.mindthegraph.com.

The I*_f_* current is modulated by the autonomic nervous system in the heart. Sympathoexcitation, characterized by an increased norepinephrine level, is a prominent feature of heart failure [91]. Treatment with ivabradine reduced circulating plasma norepinephrine and epinephrine, possibly due to increased norepinephrine reuptake 1 in the sympathetic ganglion, as demonstrated in a rat model of heart failure (Table 4) [11,65]. The gene expression of β1-adrenergic receptor in the left ventricle was also downregulated [11], which indicates attenuated sympathetic innervation by ivabradine. Reduced norepinephrine levels in the left ventricle and right and left atria accompanied by increased expression of tyrosine hydroxylase were also observed in an animal model of heart failure following treatment with ivabradine [11]. Tyrosine hydroxylase is a rate-limiting enzyme in catecholamine synthesis, and its activity is inhibited by negative feedback [92,93]. The increase in enzyme expression indicates that there is a lack of negative feedback due to low levels of catecholamines (Figure 2). 

In addition to its effect on heart rate, epinephrine can trigger a hypertrophic response in the cardiomyocytes [94]; therefore, reducing the level of the neurotransmitter is beneficial for preventing cardiac remodeling. The reduction in norepinephrine levels by ivabradine was further supported by the decreased level of the neurotransmitter and its metabolite, normetanephrine, in urine [11]. In contrast, acetylcholine, which has the opposite effect of norepinephrine (i.e., reducing heart rate) is augmented in the right atrium [11]. Therefore, ivabradine therapy blocks sympathetic overactivation by suppressing the synthesis, release, and metabolism of catecholamines in the heart. The potential effects of ivabradine on the activation of G-protein-coupled receptor kinase 2 (formerly known as β-adrenoceptor kinase) and cAMP-dependent protein kinase, two enzymes involved in catecholamine signaling, should be studied to better understand the effects of ivabradine on sympathetic excitation in the heart. Ivabradine may also modulate connexin 43, a gap junction protein that is present in the heart and mediates the communication between cells via action potentials in the heart [95]. This aspect should also be investigated.

In addition to catecholamines, activation of the renin–angiotensin–aldosterone system also negatively affects the heart [96]. The activity of angiotensin-converting enzyme (ACE), which converts angiotensin (Ang) I into Ang II, is elevated in cardiac hypertrophy and damaged hearts [97,98]. Ang II binds to Ang II type 1 receptor (AT_1_R) to exert its effects on the cardiovascular system. Ivabradine decreases the protein and gene expression of left ventricular ACE and AT_1_R without affecting endothelin 1 (ET-1) [11,63]; however, another study [53] was unable to demonstrate similar protective effects. ET-1 is a hypertrophic response stimulator and an inhibitor of norepinephrine reuptake 1 [99,100]. The drug has no effect on serum levels of Ang I, Ang II, Ang III, Ang IV, Ang 1–5, or Ang 1–7 [53,62]; however, it reduces the ratio of Ang 1–5 to Ang 1–7 [53]. Ang II [101] and Ang III [102] promote cardiac remodeling, while Ang IV [103], Ang 1–5 [104], and Ang 1–7 [105] exhibit cardioprotective effects. Serum renin and aldosterone were unaffected by treatment with ivabradine [53]. These findings demonstrate that ivabradine may regulate the renin–angiotensin–aldosterone system at the translational level. Further studies should be conducted to explore the possible impact of ivabradine on Wnt/β-catenin signaling, which has been shown to activate the cardiac renin–angiotensin–aldosterone system [106].

## 5. Effects on Myocardial Fibrosis

Myocardial fibrosis is a characteristic feature of heart failure. It appears due to disproportionate production and degradation of the extracellular matrix in cardiomyocytes, which occurs during inflammation. Degradation of the extracellular matrix by MMPs is triggered during injury repair following an insult to the heart [55,74]. Ivabradine alleviated left and right ventricular fibrosis in experimental heart failure by reducing the expression of collagen type 1 and 3, which are mainly produced by myofibroblasts and are the major constituents of the extracellular matrix (Table 5) [11,12,13,15,56,58,72,107]. Left ventricular hydroxyproline content, the main component of collagen, was also decreased following ivabradine treatment [53,54,62]. The drug reduced the expression of MMP-9 but increased the expression of MMP-2 (Figure 2) [56,71,74]. MMP-2 is synthesized constitutively, while MMP-9 expression is increased upon inflammatory stimuli [108]. Therefore, ivabradine may inhibit the MMP-9-associated inflammatory response that would have detrimental effects to the heart. A buildup of extracellular matrix material, primarily collagen, in the myocardial extracellular interstitial space may distort cardiac structure and impede its ability to contract [109].

Extracellular matrix metalloproteinase inducer (EMMPRIN) is required for MMP-dependent extracellular metalloproteinase disintegration. Ivabradine exerts cardioprotective effects by downregulating EMMPRIN and stabilizing the caveolin-3/EMMPRIN complex, resulting in reduced activity of EMMPRIN and breakdown of the extracellular matrix [74]. The drug also downregulates tissue inhibitor of metalloproteinase 2 (TIMP-2) [56], a protein that governs the activity of MMPs [110]. 

Growth factors are mediators of tissue repair. Transforming growth factor β1 (TGF-β1) and connective tissue growth factor (CTGF) are among the growth factors involved in myocardial fibrogenesis. Upon activation by cardiac insult, active TGF-β1 binds to its receptor (TGFR) to stimulate collagen production via the small mothers against decapentaplegic (Smad) signaling pathway [111]. TGF-β1 promotes the conversion of fibroblasts to myofibroblasts, defined by the presence of the highly contractile protein α-smooth muscle actin (α-SMA) [112]. Ivabradine treatment in animal models of heart failure decreased the expression of growth factors, α-SMA [12,56,58,66], and phosphorylated Smad2/3 [72]. This implies that ivabradine can prevent the transformation of fibroblasts by inhibiting profibrotic signaling, thereby reducing myocardial fibrosis. The antifibrotic effects of ivabradine were also observed in in vitro experiments [56,58], indicating that the effects were independent of I*_f_* current suppression. The effects are believed to occur through inhibition of the phosphatidylinositol 3-kinase/protein kinase/mammalian target of rapamycin complex 1/protein S6 kinase beta-1 (PI3K/Akt/mTOR/p70S6K) pathway [15]. Activation of this pathway promotes myocardial protein synthesis [15]. Consequently, ivabradine impedes the development of myocardial fibrosis.

Other signaling pathways, such as hypoxia-induced mitogenic factor-interleukin (HIMF-IL6), Ca^2+/^CaMKII-signal transducers and activators of transcription 3 (STAT3), Wnt/β-catenin, and peroxisome proliferator-activated receptor gamma (PPARγ) pathways [113,114], which are related to myocardial fibrogenesis, should also be explored. Among these pathways, PPARγ prevents fibrogenesis, while the others activate it. Ivabradine may modulate these pathways.

## 6. Effects on Biogenesis, Autophagy, and Apoptosis

Energy metabolism is crucial for maintaining the function of a myocardium. Impaired energy production occurs due to mitochondrial dysfunction in the remodeled heart [115]. Only one study explored the effects of ivabradine on energy metabolism. Ceconi et al. [14] reported that ivabradine administration (10 mg/kg/day) for 90 days restored cardiac energy metabolism in an animal model of cardiac remodeling, evidenced by increased creatine phosphate and energy charge (Table 6). Creatine phosphate serves as an energy depot for rapid ATP generation [116]. As previously mentioned, calcium is required for mitochondrial ATP production. However, exorbitant calcium content in the mitochondria is hazardous due to elevated oxidative stress leading to destruction of mitochondrial membrane potential and permeability transition pore, the opening of which would drive the depletion of ATP [80,117]. Ivabradine may modulate these parameters.

Autophagy is a cellular mechanism used to preserve metabolic processes by recycling cellular components in the heart to maintain its homeostasis and function. After autophagy is initiated, the PI3K complex is required to form a phagofore. Beclin-1 is one of the subunits in the complex [118] that promotes the maturation of autophagosomes and cargo (organelle) recruitment [119]. The elongation of the phagofore is triggered by a complex composed of autophagy-related 5 (ATG5), ATG12, and ATG16L, together with microtubule-associated protein light chain 3 II (LC3II) to form the autophagosome (Figure 2) [118]. p62 protein identifies cellular waste for removal via lysosomal sequestration before its attachment to LC3II during autophagosome formation [120].

Only one study investigated the effect of ivabradine on autophagy in a heart failure model (Table 6) [67]. The drug augmented the expression of autophagy-associated factors, including beclin-1, ATG5, ATG7, and LC3II, and decreased p62 protein levels in coronary-artery-ligation-induced cardiac remodeling in rats [67]. Cell death is increased when autophagy is suppressed (Figure 2) [121], indicating the cardioprotective effects of ivabradine regarding autophagy enhancement. It also repressed the expression of phosphorylated mammalian target of rapamycin (p-mTOR), phosphorylated PI3K, phosphorylated Akt, and phosphorylated p70S6K [67]. The findings suggest that ivabradine augments autophagy via suppression of the PI3K/Akt/mTOR/p70S6K signaling pathway. 

Apoptosis, or programmed cell death, is increased in cardiac hypertrophy. However, few studies have been conducted to investigate the role of ivabradine in this process. Yu et al. [15] demonstrated that ivabradine (10–80 mg/kg/day for 4 weeks) increased the expression of caspase 3 (a pro-apoptotic marker) and decreased the expression of cleaved caspase 3 (active form) in transverse-aortic-constriction-induced cardiac hypertrophy in mice (Table 6). The findings suggest that ivabradine prevents apoptosis by blocking the activation of caspase 3. Moreover, the drug (20 and 40 mg/kg/day for 12 weeks) was reported to diminish apoptotic events by reducing DNA fragmentation detected by terminal deoxynucleotidyl transferase dUTP nick-end labeling (TUNEL) assay in the cardiomyocytes of diabetic cardiomyopathic mice [13,107]. It is believed that ivabradine attenuates apoptosis by suppressing the PI3K/Akt/mTOR pathway [15].

The studies thus far have examined the impacts of ivabradine on myocardial apoptosis. Studies on the potential effects of the drug on mitochondrial apoptosis are lacking. Mitochondria are the “cell powerhouse” that supply energy to the cell. Impairment of mitochondrial function causes cellular dysfunction and promotes the development of cardiac remodeling. Mitochondrial respiration complex I and IV activity [122] and the survivor activating factor enhancement signaling pathway, which promotes cardiomyocyte survival, have been demonstrated to be affected in cardiac remodeling [123]. The effects of ivabradine on the mitochondrial function and apoptosis could be further explored.

## 7. Effects on Inflammation and Oxidative Stress

Inflammation plays an important role in the pathogenesis of many diseases, and it can induce cardiac remodeling [124]. Inflammation is elevated in patients with heart failure and animal models of cardiac remodeling. Many inflammatory indicators are inhibited following treatment with ivabradine. In subjects with cardiomyopathy who were treated with ivabradine, inflammatory biomarkers, namely tumor necrosis factor α (TNFα), growth-differentiation factor 15 (GDF-15), heart-type fatty acid binding protein (H-FABP), and interleukin 6 (IL-6), were attenuated (Table 7) [32,34,125]. The increase in inflammatory biomarkers was shown to correlate with the severity of disease in chronic heart failure patients [126], suggesting that the inflammation-suppressing effects of ivabradine may be translated to alleviation of heart failure symptoms in patients.

Comparable findings were demonstrated in animal studies. The gene expression and levels of inflammatory mediators, namely *TNFα*, *IL-6*, *IL-1β*, a number of inflammatory nuclei, and plasma cyclophilin A (CyPA), were mitigated by ivabradine (0.3–20 mg/kg/day for 7–84 days) in various models of experimental cardiac remodeling (Table 7) [13,67,70,71]. However, ivabradine increased CyPA expression in cardiac necrotic sites, driving increased CyPA binding to low-glycosylated EMMPRIN that resulted in decreased expression of MMP-9 [71]. High-glycosylated EMMPRIN promotes MMP production, while the low-glycosylated form has no effect on MMP synthesis [127]. Therefore, ivabradine may elicit its protective effect through the extracellular matrix-degrading protein via its modulation of the inflammatory response of CyPA.

TNFα is an inflammatory biomarker that can trigger the activation of the mitogen-activated protein kinase (MAPK) signaling pathway [128]. MAPK is a family of kinases, namely p38 MAPK, c-Jun N-terminal kinase (JNK), and extracellular signal-regulated kinase [129]. Studies have demonstrated that ivabradine prevents the activation of JNK and p38 without affecting blood glucose in diabetic animals with cardiac remodeling [13,56]. JNK and p38 activation is reported in many studies investigating cardiac hypertrophy [101,130].

TNFα can also induce the activation of the nuclear factor-kappa B (NF-κB) signaling pathway [131], which requires inhibitor of NF-κB kinase (IKK) subunits α and β. IKKβ governs the activation of the pathway via phosphorylation of nuclear factor of kappa light polypeptide gene enhancer in B-cells inhibitor (IκB). In contrast, IKKα is needed for the activation of an alternative pathway of NF-κB, which is independent of TNFα [132]. Ivabradine at 20 and 40 mg/kg/day for 12 weeks inhibited the activation of IKKα/β and IκBα (Figure 2) [107]. Furthermore, ivabradine-mediated inhibition of MAPK and NF-κB signaling was observed in cell cultures [56,107], confirming that the effects surpassed I*_f_* current suppression. Involvement of the NF-κB signaling pathway in cardiac hypertrophy has been reported elsewhere [133].

The effects of ivabradine on oxidative stress have not been extensively explored. Only one study reported that ivabradine at 10 mg/kg/day for 12 weeks augmented myocardial superoxide dismutase (SOD) protein expression in rats with ligated abdominal aorta for heart failure induction [12]. SOD is a frontline antioxidant enzyme that converts superoxide anion into hydrogen peroxide and water [129]. SOD augmentation by ivabradine was associated with a reduction in growth factors (CTGF and *TGF-β1*) and collagen expression [12], demonstrating the ability of the drug to decrease myocardial fibrosis via its antioxidant activity.

Taken together, these findings suggest that ivabradine possesses both anti-inflammatory and antioxidant properties. The drug may regulate the nucleotide-binding domain, leucine-rich repeat family pyrin domain containing receptor 3 (NLRP3) inflammasome [134], or the interaction of nuclear factor erythroid 2-related factor 2 (Nrf2) and Kelch-like ECH-associated protein 1 (Keap1) [135], which are activated in response to pressure-overload-induced cardiac remodeling in rodents. The latter is involved in the amelioration of oxidative stress.

## 8. Effects on Cardiac Structure

As previously described, the pathogenesis of cardiac remodeling and consequent heart failure involves many signaling pathways, such as the renin–angiotensin–aldosterone system, Smad, and PI3K/Akt/mTOR/p70S6K pathway. These events eventually cause changes to heart structure and size, which are commonly manifested by ventricular wall thickening, leading to enlargement of cardiac size. These changes ultimately alter cardiac function. Ivabradine therapy has been demonstrated to decrease B-type natriuretic peptide (BNP) and N-terminal proBNP (NT-proBNP)—cardiac dysfunction biomarkers—in patients with heart failure (Table 8) [20,23,29,34]. BNP is released into the circulation by the myocardial ventricles in response to volumetric stretch of the heart [136]. The release of BNP and NT-proBNP is associated with increased heart mass [129,137]. However, the effect of ivabradine on heart mass, especially left ventricular mass, in patients with heart failure has not been extensively studied. Only Bonadei et al. [41] reported that ivabradine reduced left ventricular end-systolic diameter (LVESD), indicating the ability of the drug to reduce left ventricular thickening.

In animal studies, ivabradine attenuated cardiomyocyte size, left ventricular and left atrial mass, and the ratio of heart weight to body weight (or to tibial length) [11,14,15,54,58,63,72,73], which was in line with a reduction in the expression of atrial natriuretic peptide (*ANP*), another type of natriuretic peptide (Table 8) [11,14]. The amelioration of cardiac structure by ivabradine in different animal models of heart failure was investigated using echocardiography. The drug attenuated the progression of left ventricular cardiac remodeling, as evidenced by decreased left ventricular diastolic (LVDD) and systolic (LVSD) dimension, LVESD, posterior wall thickness at diastole (LVPWd), and internal diameter at diastole (LVIDd) and systole (LVIDs) [11,15,65,73]. Furthermore, it decreased interventricular septal thickness at diastole (IVSd) and systole (IVSs) [15,58]. The beneficial effects of ivabradine were also appreciated in the left atrium dimension [11,63,73] and right ventricular structure, evidenced by a reduction in the end-diastolic diameter (RVEDD) [72]. Despite the varied findings, ivabradine produces potential beneficial effects on cardiac structure, possibly arising from its inhibitory impact on the I*_f_* current. A decrease in heart rate reduces myocardial workload, improves myocardial structure, and subsequently improves myocardial function.

## 9. Conclusions and Aspects for Future Studies

Increasing evidence has established that ivabradine alleviates the symptoms of heart failure in patients. The comparative beneficial effects of ivabradine in terms of its mechanism of action were also manifested in animal studies. In summary, ivabradine exerts cardioprotective effects via its selective inhibition of I*_f_* current, resulting in heart rate reduction and amelioration of heart-failure-associated symptoms in patients. It exhibits pleiotropic antiremodeling in animal studies through several mechanisms including antifibrotic, anti-inflammatory, antioxidant, and antiapoptotic effects. It also augments autophagy and mitochondrial bioenergetics in the animals. Figure 2 outlines the molecular mechanisms of ivabradine in heart failure. More clinical studies are needed to further clarify certain aspects of the effects of ivabradine in patients, such as myocardial fibrosis, which can be investigated using cardiac magnetic resonance imaging. Parameters, such as extracellular volume fraction, extracellular matrix volume, total myocardial volume, and cellular volume, can be measured using this technology. Moreover, myocardial energetics calculated as the phosphocreatine-to-ATP ratio can be determined [138]. The clinical effects of ivabradine on cardiac structure should also be investigated extensively to confirm the findings observed in animal studies.

In terms of its mechanism of action, more research should be conducted in animal studies (in vivo, in vitro, or ex vivo) to examine the effects of ivabradine on cellular calcium handling (e.g., transient receptor potential canonical channels), mitochondrial function and biogenesis (e.g., sirtuin 3), autophagy (e.g., FUN14 domain containing 1 and BCL2-interacting protein 3 like), inflammation (e.g., calcineurin-nuclear factor of activated T-cell), and oxidative stress signaling mechanisms (e.g., AMP-activated protein kinase/Nur77). Its potential antifibrotic effects on modulating zinc finger transcription factor GATA-binding protein 4 (GATA4) should also be investigated.

Based on its ability to inhibit I*_f_* current, ivabradine could be considered hypothetically to treat tachycardia in inappropriate sinus syndrome, postural orthostatic tachycardia syndrome, and refractory junction ectopic tachycardia. Its antiremodeling potential might be of benefit in hypertensive heart protection. 

## Figures and Tables

**Table 1:** Effects of ivabradine therapy on clinical outcomes in patients with heart failure.

Subjects	Dose of Ivabradine	Type of Study	Findings	Reference
Patients with HF (LVEF < 40%, HR > 70 bpm) (n = 37)	2.5–7.5 mg, b.i.d. for >12 months	Retrospective cohort study	↓ risk of hospitalization ↓ number of hospitalizations ↔ length of hospitalization ↔ death rate	[16]
Moderate-to-severe HF patients with HR > 70 bpm (n = 3241) (SHIFT study)	Started at 5 mg b.i.d. and titrated to 7.5 mg b.i.d. or 2.5 mg b.i.d.	Randomized, double-blind, placebo-controlled, parallel-group, multicenter clinical trial	↓ event rates in patients with 0 or 3+ comorbidities ↓ HF hospitalization	[17]
Hemodynamically stable acute HF patients (n = 63)	Started at 5 mg daily, followed by 10 mg daily for >90 days	Retrospective cohort	↓ length of hospitalization ↓ rehospitalization ↓ high dose of β-blockers ↓ NYHA class	[18]
Moderate-to-severe HF patients with HR > 77 bpm (n = 208) (SHIFT study)	Started at 5 mg b.i.d. and titrated to 7.5 mg b.i.d. or 2.5 mg b.i.d. for 31–35 months	Randomized, double-blind, placebo-controlled, parallel-group, multicenter clinical trial	↓ NYHA class ↑ Global self-assessment improvement ↑ Global assessment improvement (physician perspective) ↑ Health-related quality of life ↓ all-cause cardiovascular death ↓ all-cause hospitalization ↓ all-cause mortality	[19]
Patients with chronic HF (n = 767) (RELIf-CHF study)	5 mg b.i.d. and titrated to 7.5 mg or 2.5 mg b.i.d. for 12 months	Observational follow-up study	↓ NYHA class ↓ decompensation ↓ HF hospitalizations ↑ general health ↑ QoL	[20]
Moderate-to-severe HF patients with HR < 75 (n = 1188) and >75 bpm (n = 2052) (SHIFT study)	5 mg b.i.d. titrated to 7.5 mg b.i.d. for a median follow-up of 22.5 months	Randomized, double-blind, placebo-controlled, parallel-group, multicenter clinical trial	In HR > 75 bpm group: ↓ cardiovascular death ↓ death from HF ↓ hospitalization In HR < 75 bpm group: ↔ cardiovascular death ↔ death from HF ↔ hospitalization	[21]
Hospitalized HF patients in the SHIFT study (n = 514)	Started at 5 mg b.i.d. and titrated to 7.5 mg b.i.d. or 2.5 mg b.i.d. for 3 months	Randomized, double-blind, placebo-controlled, parallel-group, multicenter clinical trial	↓ all-cause hospitalization at 1, 2, and 3 months ↔ hospitalization due to cardiovascular causes at all time-points ↔ death rate	[22]
Acute HF patients with inflammatory rheumatic disease (n = 12)	2.5 mg/d b.i.d. titrated to 5 mg/d b.i.d. for 2 weeks	Retrospective observational study	↓ NYHA class	[23]
Moderate-to-severe HF patients with HR > 70 bpm plus angina pectoris (n = 1085) (SHIFT and SIGNIFY studies)	Started at 5 mg b.i.d. and titrated to 7.5 mg b.i.d. or 2.5 mg b.i.d. for 31-35 months	Randomized, double-blind, placebo-controlled, parallel-group, multicenter clinical trial	SHIFT study: ↔ Composite primary end point ↔ Cardiovascular death ↔ First hospitalization due to worsening HF SIGNIFY study: ↔ Composite primary end point ↔ Cardiovascular death ↔ non-fatal MI	[24]
Moderate-to-severe HF patients (HR > 70 bpm) with prior mineralocorticoid receptor antagonist (MRA) (n = 1981) (SHIFT study)	Started at 5 mg b.i.d. and titrated to 7.5 mg b.i.d. or 2.5 mg b.i.d. for 31–35 months	Randomized, double-blind, placebo-controlled, parallel-group, multicenter clinical trial	Compared to the MRA group at baseline: ↔ Composite primary end point ↔ Cardiovascular death ↔ HF death	[25]
Moderate-to-severe HF patients (HR > 70 bpm) with diabetes (n = 973) (SHIFT study)	Started at 5 mg b.i.d. and titrated to 7.5 mg b.i.d. or 2.5 mg b.i.d. for 31–35 months	Randomized, double-blind, placebo-controlled, parallel-group, multicenter clinical trial	↔ Outcomes of different treatments (ivabradine vs. placebo; insulin vs. non-insulin) In diabetic and non-diabetic patients: ↓ hospitalization for worsening HF ↓ cardiovascular hospitalization In non-diabetic patients: ↓ all-cause hospitalization	[26]
Patients with HFpEF (n = 84) (EDIFY study)	Started at 5 mg b.i.d. and titrated to 7.5 mg b.i.d. or 2.5 mg b.i.d. for 8 months	Randomized, double-blind, placebo-controlled, multicenter clinical trial	↔ 6MWT	[27]
Acute decompensated HFrEF patients (n = 292)	Not given. Follow-up for 1 year after discharge	Retrospective study	↓ cardiovascular death ↓ all-cause mortality ↓ rehospitalization ↓ NYHA class	[28]
Patients with systolic chronic HF (n = 98)	Started at 5 mg b.i.d. and titrated to 7.5 mg b.i.d. or 2.5 mg b.i.d. for 6 months	Open-label, blinded, parallel-group, interventional, prospective-cohort study	↓ NYHA class	[29]
Moderate-to-severe HF patients (HR > 70 bpm) with left bundle branch block (n = 467) (SHIFT study)	Started at 5 mg b.i.d. and titrated to 7.5 mg b.i.d. or 2.5 mg b.i.d. for 31-35 months	Randomized, double-blind, placebo-controlled, parallel-group, multicenter clinical trial	↔ primary end point ↔ cardiovascular death ↔ HF hospitalization ↔ all-cause death	[30]
Patients with chronic HF (n = 110) (APULIA study)	5 mg b.i.d. for a month	Multicentric observational study	↓ HR ↑ physical functioning ↑ physical role functioning ↑ emotional role functioning ↑ mental health scale	[31]
Patients with cardiomyopathy (n = 33)	5 mg b.i.d. for 3 months and 7.5 mg b.i.d. for 3 months	Observational study	↓ NYHA class ↑ general health ↑ social activity ↑ physical health ↑ emotional health	[32]
Hospitalized patients with acute decompensated systolic heart failure (n = 10)	Started at 5 mg b.i.d. and titrated to 7.5 mg b.i.d. or 2.5 mg b.i.d. until discharged	Observational, open-label, longitudinal, and retrospective study	↓ NYHA class	[33]
Patients with HF (n = 10)	5 mg b.i.d. and titrated to 7.5 mg b.i.d. for 6 months	Randomized, double-blind study	↓ NYHA class ↑ QoL	[34]
Patients with chronic HF (n = 1873)	5 mg b.i.d. and titrated to 7.5 mg or 2.5 mg b.i.d. for 4 months	Observational and longitudinal study	↓ NYHA class ↓ decompensation	[35]
Children with dilated cardiomyopathy (n = 74)	0.02 mg/kg b.i.d. (6–12 months old) or 0.05 mg/kg b.i.d. (1–18 years old) or 2.5 mg b.i.d. (>40 kg bw) and titrated for 12 months.	Randomized, double-blind, placebo-controlled, phase II/III clinical trial	↑ PedQL ↔ NYHA class	[36]

b.i.d., twice daily; bw, body weight; HF, heart failure; HFpEF, heart failure with preserved ejection fraction; HFrEF, heart failure with reduced ejection fraction; HR, heart rate; LVEF, left ventricular ejection fraction; MRA, mineralocorticoid receptor antagonist; NYHA, New York Heart Association; PedQL, pediatric quality of life inventory; QoL, quality of life; 6MWT, 6 min walking test; ↔, no difference; ↓, reduced; ↑, increased.

**Table 2:** Effects of ivabradine on cardiac function in human studies.

Subjects	Dose of Ivabradine	Type of Study	Findings	Reference
Hospitalized patients with severe CHF (n = 10)	Infusion at 0.1 mg/kg for 90 min, followed by 0.05–0.075 mg/kg for 90 min	Single-center open-label phase II clinical trial	At 4 h: ↓ HR, ↑ SV ↑ LV systolic work	[40]
Hemodynamically stable acute HF patients (n = 63)	Started at 5 mg daily, followed by 10 mg daily for > 90 days	Retrospective cohort	↓ HR, ↑ LVEF ↔ SBP, ↔ DBP	[18]
Patients with chronic HF (n = 1873)	5 mg b.i.d. and titrated to 7.5 mg or 2.5 mg b.i.d. for 4 months	Observational and longitudinal study	↑ LVEF	[35]
Acute decompensated HFrEF patients (n = 292)	Not given. Follow-up for 1 year after discharge	Retrospective study	↓ HR ↔ SBP, ↔ LVEF	[28]
Moderate-to-severe HF patients with HR < 75 (n = 1188) and >75 bpm (n = 2052) (SHIFT study)	5 mg b.i.d. titrated to 7.5 mg b.i.d. for a median follow-up of 22.5 months	Randomized, double-blind, placebo-controlled, parallel-group, multicenter clinical trial	In HR > 75 bpm group: ↓ HR In HR < 75 bpm group: ↔ HR	[21]
Moderate-to-severe HF patients with HR > 70 bpm (n = 298) (SHIFT study)	Started at 5 mg b.i.d. and titrated to 7.5 mg b.i.d. or 2.5 mg b.i.d. for 8 months	Randomized, double-blind, placebo-controlled, parallel-group, multicenter clinical trial	↓ office HR ↓ 24-HR ↓ HR awake ↓ HR asleep	[42]
Patients with chronic HF (n = 30)	5 mg b.i.d. for 4 months	Cross-sectional	↓ LVEDV, ↓ LVESV ↑ LVEF, ↑ SV, ↑ Ees ↓ VAC	[41]
Acute HF patients with inflammatory rheumatic disease (n = 12)	2.5 mg/d b.i.d. titrated to 5 mg/d b.i.d. for 2 weeks	Retrospective observational study	↓ HR ↑ LVEF	[23]
Moderate-to-severe HF patients with HR > 77 bpm (n = 208) (SHIFT study)	Started at 5 mg b.i.d. and titrated to 7.5 mg b.i.d. or 2.5 mg b.i.d. for 31–35 months	Randomized, double-blind, placebo-controlled, parallel-group, multicenter clinical trial	↓ LVESVI, ↓ LVESV, ↓ LVEDVI, ↓ LVEDV, ↑ LVEF	[19]
Patients with HFpEF (n = 84) (EDIFY study)	Started at 5 mg b.i.d. and titrated to 7.5 mg b.i.d. or 2.5 mg b.i.d. for 8 months	Randomized, double-blind, placebo-controlled, multicenter clinical trial	↓ HR ↔ E/e′, ↔ E, ↔ Ea, ↔ Ees, ↔ Ea/Ees ↔ Total mitral flow duration ↔ Mitral flow integral time velocity ↔ Lateral e′, ↔ Septal e′ ↔ Mean of lateral and septal e′ ↔ LVEDV, ↔ SV, ↔ LAVI	[27]
Male patients with chronic HF (n = 22)	5 mg b.i.d. and titrated to 7.5 mg for 6 months	Longitudinal study	↓ HR ↔ SBP, ↔ DBP, ↔ LVEF	[43]
Patients with systolic chronic HF (n = 98)	Started at 5 mg b.i.d. and titrated to 7.5 mg b.i.d. or 2.5 mg b.i.d. for 6 months	Open-label, blinded, parallel-group, interventional, prospective-cohort study	↓ HR	[29]
Patients with systolic HF (n = 43)	Started at 5 mg b.i.d. and titrated to 7.5 mg b.i.d. or 2.5 mg b.i.d. for 3 months	Longitudinal study	↓ HR ↔ SBP, DBP ↔ LVEDV, LVESV, LVEF, ↔ E/A, ↓ E/E′ ↓ LA Vmax, ↓ LA Vp ↔ LA Vmin ↔ LA passive emptying volume and fraction ↓ LA active emptying volume and fraction ↓ PA lateral, septum, and tricuspid ↓ PA lateral–PA tricuspid ↔ PA lateral–PA septum ↓ PA septum–PA tricuspid ↓ interatrial conduction delay ↔ left intra-atrial conduction delay ↓ right intra-atrial conduction delay	[44]
Moderate-to-severe HF patients (HR > 70 bpm) (n = 143) (SHIFT study)	Started at 5 mg b.i.d. and titrated to 7.5 mg b.i.d. or 2.5 mg b.i.d. for 8 months	Randomized, double-blind, placebo-controlled, parallel-group, multicenter clinical trial	↓ HR, ↔ LVESP, ↑ SV ↔ Pulse pressure, ↔ MAP ↑ Total arterial compliance ↓ Ea, ↔ TPR, ↔ CO, ↔ Ees ↑ LVEF, ↔ LVESV ↔ LVEDV, ↔ Ea/Ees	[45]
Patients with cardiomyopathy (n = 33)	5 mg b.i.d. for 3 months and 7.5 mg b.i.d. for 3 months	Observational study	↓ HR, ↑ LVEF	[32]
Hospitalized patients with acute decompensated systolic heart failure (n = 10)	Started at 5 mg b.i.d. and titrated to 7.5 mg b.i.d. or 2.5 mg b.i.d. until discharged	Observational, open-label, longitudinal, and retrospective study	↓ HR, ↓ SBP ↔ DBP, ↔ MBP	[33]
Moderate-to-severe HF patients (HR > 70 bpm) with left bundle branch block (n = 208) (SHIFT study)	Started at 5 mg b.i.d. and titrated to 7.5 mg b.i.d. or 2.5 mg b.i.d. for 8 months	Randomized, double-blind, placebo-controlled, parallel-group, multicenter clinical trial	↓ LVESVI, ↓ LVEDVI ↓ LVESV, ↓ LVEDV ↑ LVEF	[46]
Patients with HF (n = 10)	5 mg b.i.d. and titrated to 7.5 mg b.i.d. for 6 months	Randomized, double-blind, double-dummy study	↑ VO_2_	[34]
Patients with chronic HF (n = 1873)	5 mg b.i.d. and titrated to 7.5 mg or 2.5 mg b.i.d. for 4 months	Observational and longitudinal study	↑ LVEF	[35]
Patients with chronic HF (n = 767) (RELIf-CHF study)	5 mg b.i.d. and titrated to 7.5 mg or 2.5 mg b.i.d. for 12 months	Observational follow-up study	↓ HR, ↑ LVEF	[20]
Patients with stable symptomatic chronic HF (n = 52)	5 mg b.i.d. and titrated to 7.5 mg 2.5 mg b.i.d. for 12 months	Observational follow-up study	↓ LVEDV, ↓ LVESV, ↑ LVEF, ↓ DT ↔ TAPSE, ↔ PASP, ↔ RV FAC, ↔ E peak, ↔ A peak, ↔ myocardial performance index ↑ systolic velocity ↑ Early diastolic velocity ↓ Late diastolic velocity ↔ RV IVV, ↔ RV IVA ↑ RV GLS, ↑ RV LS ↑ RV LSRS, ↑ RV LSRE ↑ RV LSRA	[47]
Children with dilated cardiomyopathy (n = 74)	0.02 mg/kg b.i.d. (6–12 months old) or 0.05 mg/kg b.i.d. (1–18 years old) or 2.5 mg b.i.d. (>40 kg bw) and titrated for 12 months.	Randomized, double-blind, placebo-controlled, phase II/III clinical trial	↓ HR, ↑ LVEF	[36]

b.i.d., twice daily; bw, body weight; CHF, congestive heart failure; CO, cardiac output; DBP, diastolic blood pressure; DT, deceleration time; E, early diastolic mitral inflow velocity; E′, early diastolic mitral annular velocity; Ea, arterial elastance; E/A, early-to-late diastolic mitral inflow velocity; E/e′, ratio of peak early diastolic mitral flow velocity to the mean of annular lateral and septal velocities; Ees, left ventricular end-systolic elastance; FAC, fractional area change; GLS, global longitudinal strain; HF, heart failure; HR, heart rate; IVA, myocardial acceleration during isovolumic contraction; IVV, peak myocardial velocity during isovolumic contraction; LA, left atrium; LAVI, left atrial volume index; LS, longitudinal strain; LSRA, longitudinal strain rate diastolic late filling; LSRE, longitudinal strain rate diastolic early filling; LSRS, systolic longitudinal strain rate; LV, left ventricle; LVEDV, left ventricular end-diastolic volume; LVEF, left ventricular ejection fraction; LVESP, left ventricular end-systolic pressure; LVESV, left ventricular end-systolic volume; LVESVI, left ventricular end-systolic volume index; MAP, mean arterial pressure; MBP, mean blood pressure; PA, the interval from the onset of P wave to appearance of the late diastolic wave in Doppler imaging; PASP, pulmonary artery systolic pressure; RV, right ventricle; SBP, systolic blood pressure; SV, stroke volume; TAPSE, tricuspid annular plane systolic excursion; TPR, total peripheral resistance; Vmax, maximum volume at the end-systolic phase; Vmin, minimum volume at the end-diastolic phase; VAC, ventricular-arterial coupling; VO_2_, peak oxygen consumption; Vp, volume before P wave; ↔, no difference; ↓, reduced; ↑, increased.

**Table 3:** Effects of ivabradine on cardiac function in animal studies.

Models	Dose and Duration of Ivabradine	Findings	Reference
Surface ECG recordings and transesophageal electrophysiological study in female C57BL/10 mice	Single dose of 10 mg/kg (i.p.)	↓ HR ↑ QRS duration ↔ QR duration ↑ QT1 intervals ↑ QT2-P intervals ↑ S2Q2 intervals	[50]
Chronic-hypertension-induced cardiac hypertrophy in pigs	1 mg/kg/d infusion for 28 days	↓ HR, ↑ SV, ↑ LVEDP ↑ LV twist, ↔ LV twisting rate ↑ LV untwisting rate ↑ LV untwisting velocity at MVO ↔ LV apical rotation ↑ LV basal rotation ↑ untwist during isovolumic relaxation time	[51]
Experimental chronic- hypertension-induced cardiac remodeling in pigs	1 mg/kg (i.v. bolus, single)	↓ HR, ↔ CO ↔ dp/dt_max_, ↔ LV pressure ↑ LV end-diastole internal diameter ↑ LV end-systole internal diameter ↑ LV relaxation filling ↑ LV early filling ↑ LV peak early filling rate	[52]
Experimental hypertension- induced cardiac remodeling in SHR	10 mg/kg/d in drinking water for 6 weeks	↓ HR, ↔ SBP, ↑ LVEF ↑ LVFS, ↓ E/A, ↓ E/Em	[53]
Isoproterenol-induced heart failure in rats	10 mg/kg/d (p.o.) for 6 weeks	↓ HR	[54]
Isoproterenol-induced heart failure in rats	10 mg/kg/d (p.o.) for 14 days	↓ HR	[55]
Diastolic-dysfunction-induced heart failure in diabetic mice	20 mg/kg/d in drinking water for 4 weeks	↓ HR, ↑ E/A, ↓ EDT ↑ −dp/dt_min_, ↓ Tau, ↓ IVRT	[56]
Diabetic cardiomyopathy in mice	20 mg/kg/d (p.o.) for 12 weeks	↓ HR, ↑ LVEF	[13]
Myocardial I/R-induced cardiac remodeling in rats	10 mg/kg/d (p.o.) for 28 days	↓ HR, ↑ LVFS ↑ LVEF, ↑ delta LVEF	[57]
Experimental HFpEF in mice	10 mg/kg/d (low) and 20 mg/kg/d (high) (p.o.) for 4 weeks	High dose: ↓ HR, ↓ LVEDP, ↔ LVEF ↓ LV −dp/dt_max_, ↔ LV +dp/dt_max_, ↓ EDT, ↔ LVFS, ↓ IVRT Low dose: ↓ HR	[58]
Experimental HFrEF in mice	10 mg/kg/d and 20 mg/kg/d (p.o.) for 8 weeks	High dose: ↓ HR, ↓ LVEDP, ↓ IVRT ↓ LV −dp/dt_max_ ↑ LV +dp/dt_max_ ↓ EDT, ↑ LVEF, ↑ LVFS Low dose: ↓ HR	[58]
Post-MI-induced heart failure in rats	10 mg/kg/min (via osmotic pump) for 2 weeks	↓ HR, ↑ CO, ↑ SV, ↔ LVEF ↔ LV +dp/dt ↔ LV −dp/dt ↔ LVEDP	[59]
Myocardial I/R-induced cardiac remodeling in pigs	0.3 mg/kg (i.v.)	↓ HR, ↑ SV, ↓ CO, ↑ CVP ↔ MAP ↔ systemic arterial pressure ↔ pulmonary arterial pressure	[60]
Hypertension-induced heart failure in rats	10 mg/kg/d in drinking water for 10 weeks	↓ HR, ↔ SBP, ↓ E/A, ↓ E/E′ ↑ LVFS, ↑ LVEF	[11]
MI-induced cardiac remodeling in rats	10 mg/kg/d in drinking water for 8 weeks	↓ HR, ↑ LVEF, ↓ LVEDP ↑ LVDP, ↑ LV +dp/dt ↑ LV −dp/dt ↓ LV diastolic wall stress	[61]
Experimental hypertension- induced cardiac remodeling in rats	10 mg/kg/d in drinking water for 4 weeks	↓ HR, ↓ SBP, ↑ LVEF ↑ LVFS	[62]
Severe post-MI chronic HF in rats	10 mg/kg/d in drinking water for 3 months	↓ HR, ↑ LVEF, ↓ LVEDP ↓ LVEDV, ↓ LVESV ↑ SV, ↔ CO	[63]
Abdominal-aorta- constriction-induced chronic heart failure in rats	10 mg/kg/d (p.o.) for 12 weeks	↓ LVEDP, ↑ LV +dp/dt ↓ L V −dp/dt	[12]
Open chest with LV post- ischemia dysfunction in pigs	Bolus infusion of 0.5 mg/kg	↓ HR, ↑ SV, ↔ CO ↑ diastolic filling time ↔ MAP, cardiac efficiency	[64]
Chronic ischemic heart failure in diabetic rats	10 mg/kg/d (i.p.) for 7 weeks	↓ HR, ↑ LVFS, ↓ LVEDP	[65]
LAD coronary-artery- ligated-induced cardiac remodeling in rats	10 mg/kg/d in drinking water for 90 days	↓ HR, ↑ LVEF, ↔ LVEDV ↔ LVESV	[14]
LAD coronary-artery- ligated-induced cardiac remodeling in rats	6–8 mg/kg/d (i.p.) for 4 weeks	↓ HR, ↑ SV, ↔ LVEDV ↔ LVESV, ↓ LVEDV/LV mass ↑ LVEF, ↓ LVEDP ↑ LV coronary reserve ↔ coronary conductance	[66]
LAD coronary-artery- ligated-induced cardiac remodeling in rats	10 mg/kg/d (i.g.) for 7 days	↑ LVSP, ↓ LVEDP ↑ +dp/dt_max_, ↓ −dp/dt_max_	[67]
Doxorubicin-induced LV dysfunction in rats	10 mg/kg (i.p.), alternate days for 2 weeks	↓ HR, ↔ MAP, ↑ +dp/dt_max_ ↑ Tau, ↑ SDNN, ↓ LF ↔ HF, ↓ LF/HF, ↑ RMSSD ↑ Total power	[68]
Pulmonary-arterial- hypertension-induced heart failure in rats	10 mg/kg/d (p.o.) for 3 weeks	↔ HR, ↑ RV S′, ↑ LV E’ ↓ RV fractional area ↓ RV IVCT, ↓ LV IVCT ↓ Time to mitral valve opening ↓ Time to RV peak radial motion ↓ Time to maximum LVSB ↓ Time to maximum TAPSE ↓ Time to tricuspid valve opening ↓ RV Tau (τ)	[69]
Hypertension-induced cardiac remodeling in SHR	1 mg/kg/d (i.p.) for 14 days	↓ HR, ↓ SBP, ↓ DBP, ↓ MAP	[70]
Transverse-aortic- constriction-induced cardiac hypertrophy in mice	10, 20, 40, and 80 mg/kg/d (i.g.) for 4 weeks	All doses: ↓ HR, ↓ LV Vols, ↑ LVEF ↑ LVFS 10 and 20 mg/kg/d: ↓ LV Vold	[15]
Myocardial I/R-induced cardiac remodeling in pigs	0.3 mg/kg for 7 days	↑ LVEF	[71]
Pulmonary-hypertension- induced cardiac remodeling in rats	10 mg/kg/d (p.o.) for 3 weeks	↓ HR, ↓ RV longitudinal ↑ RV S′, ↓ RV S:D ratio ↓ RV TDI-MPI, ↓ TDI IVRT ↓ RDI IVRT/R-R, ↑ SV, ↑ CO ↑ RV +dp/dt, ↓ RV −dp/dt ↓ RV Tau	[72]
RV pressure-loaded-induced cardiac remodeling in rats	10 mg/kg/d (p.o.) for 3 weeks	↓ HR, ↑ FAC, ↑ TAPSE ↓ RV MPI, ↓ RV S:D ratio ↓ RV longitudinal ↓ RV TDI-MPI, ↓ TDI IVRT ↓ RDI IVRT/R-R, ↑ SV, ↑ CO ↓ RV EDP, ↑ RV +dp/dt ↓ RV −dp/dt, ↓ RV Ees ↓ RV Tau	[72]
SU5416+Hypoxia-induced cardiac remodeling in rats	10 mg/kg/d (p.o.) for 3 weeks	↓ HR, ↑ FAC, ↑ TAPSE ↓ RV MPI, ↓ RV TDI-MPI ↓ TDI IVCT, ↓ TDI IVRT ↓ RDI IVRT/R-R, ↑ SV, ↑ CO ↓ RV EDP, ↓ RV Ees, ↓ RV EDPVR, ↓ RV Tau	[72]
Hyperthyroid cardiomyopathy in rats	10 mg/kg/d (p.o.) for 28 days	↓ HR, ↓ EDT, ↑ E_a_, ↓ E/E_a_ ↓ S_circ_, ↓ SR_circ_, ↓ S_long_ ↑ SR_long_, ↑ S_rad_, ↑ SR_rad_	[73]
Cardiogenic-shock-induced cardiac remodeling in pigs	0.3 mg/kg (i.v. bolus)	↓ HR, ↑ SV, ↑ LVEF	[74]

A, late diastolic mitral inflow velocity; CO, cardiac output; CVP, central venous pressure; DBP, diastolic blood pressure; ECG, electrocardiogram; +dp/dt_max_, maximal rate of rise of left ventricular pressure; −dp/dt_max_, maximal rate of fall of left ventricular pressure; E, early diastolic mitral inflow velocity; E′, early diastolic mitral annular velocity; E_a_, peak early diastolic mitral annular velocity; E/A, early-to-late diastolic mitral inflow velocity; ECG, electrocardiogram; EDP, end-diastolic pressure; EDPVR, end-diastolic pressure–volume relation; EDT, E peak deceleration time; Ees, left ventricular end-systolic elastance; Em, the maximal velocity of early diastolic wall movement wave at the level of mitral annulus; FAC, fractional area change; HF, power in high-frequency range; HFpEF, heart failure with preserved ejection fraction; HFrEF, heart failure with reduced ejection fraction; HR, heart rate; i.p., intraperitoneum; I/R, ischemia/reperfusion; i.v., intravenous; IVCT, isovolumic contraction time; IVRT, isovolumetric relaxation time; LF, power in low-frequency range; LV, left ventricle; LAD, left anterior descending; LVEDP, left ventricular end-diastolic pressure; LVEDV, left ventricular end-diastolic volume; LVEF, left ventricular ejection fraction; LVESV, left ventricular end-systolic volume; LVFS, left ventricular fractional shortening; LVSB, early diastolic left ventricular septal bowing; LVSP, left ventricular systolic pressure; MAP, mean arterial pressure; MI, myocardial infarction; MPI, myocardial performance index; MVO, mitral valve opening; p.o., per oral; RMSSD, square root of the mean squared differences of successive normal-to-normal intervals; RV, right ventricle; R-R, electrocardiogram R wave to R wave interval; S′, systolic tissue wave velocity; S_circ_, circumferential strain; SBP, systolic blood pressure; SR_circ_, circumferential strain rate; S_long_, longitudinal strain; SR_long_, longitudinal strain rate; S_rad_, radial strain; SR_rad_, radial strain rate; SBP, systolic blood pressure; S:D, ratio of systolic duration to diastolic duration; SDNN, standard deviation of all normal-to-normal intervals; SHR, spontaneous hypertensive rats; SU5416, a tyrosine kinase inhibitor; SV, stroke volume; TAPSE, tricuspid annular plane systolic excursion; Tau, relaxation time constant; TDI, tissue Doppler imaging; Vold, volume in diastole; Vols, volume in systole; ↔, no difference; ↓, reduced; ↑, increased.

**Table 4:** Effects of ivabradine on the cardiac conduction system and renin–angiotensin–aldosterone system.

Models	Dose and Duration of Ivabradine	Findings	Reference
Hypertension-induced HF in rats	10 mg/kg/d in drinking water for 10 weeks	↔ LV *HCN2* gene ↔ LV *HCN4* gene ↔ LA *HCN2* gene ↔ LA *HCN4* gene ↓ RA *HCN2* gene ↔ RA *HCN4* gene ↓ LV NE, ↓ RA NE, ↓ LA NE ↓ urine NE ↓ urine normetanephrine ↑ RA Ach, ↔ LA Ach ↓ serum NE ↓ serum epinephrine ↔ serum dopamine ↑ LV tyrosine hydroxylase protein ↑ LA tyrosine hydroxylase protein ↑ RA tyrosine hydroxylase protein ↓ LV *ACE* gene ↔ LV *ET-1* gene ↓ LV *AVP* gene ↓ LV *β1* adrenoceptor gene ↓ LV *NGF* gene	[11]
Post-MI-induced HF in rats	10 mg/kg/min (via osmotic pump) for 2 weeks	↓ HCN4 expression	[59]
Hypertension-induced cardiac remodeling in SHR	1 mg/kg/d (i.p.) for 14 days	↓ LV *HCN4* mRNA	[70]
Chronic ischemic heart failure in diabetic rats	10 mg/kg/d (i.p.) for 7 weeks	↓ plasma NE ↑ NE uptake-1 in stellate ganglion tissues	[65]
Severe post-MI chronic HF in rats	10 mg/kg/d in drinking water for 3 months	↓ LV *ACE* mRNA ↓ LV *AT_1_R* mRNA ↓ LV ACE protein ↓ LV. AT1R protein	[63]
Experimental hypertension-induced cardiac remodeling in SHR	10 mg/kg/d in drinking water for 6 weeks	↔ serum Ang 1–10 (Ang I), Ang 1–8 (Ang II), Ang 2–8 (Ang III), Ang 3–8 (Ang IV), Ang 1–7, Ang 1–5 ↓ (Ang 1–5)/(Ang 1–7) ↔ serum renin ↔ serum ACE ↔ serum aldosterone ↔ serum aldosterone/Ang II ratio	[53]
LAD coronary-artery-ligated-induced cardiac remodeling in rats	6–8 mg/kg/d (i.p.) for 4 weeks	↓ plasma Ang II ↓ LV AT_1_R protein ↔ LV bradykinin protein	[66]
Experimental hypertension-induced cardiac remodeling in rats	10 mg/kg/d in drinking water for 4 weeks	↔ serum aldosterone ↔ serum renin ↔ serum Ang 1–10 (Ang I), Ang 1–8 (Ang II), Ang 2–8 (Ang III), Ang 3–8 (Ang IV), Ang 1–7, Ang 1–5	[62]

Ach, acetylcholine; Ang, angiotensin; ACE, angiotensin-converting enzyme; AT_1_R, Ang II type 1 receptor; AVP, arginine vasopressin; ET-1, endothelin-1; HCN, hyperpolarization-activated cyclic nucleotide-gated channels; HF, heart failure; i.p., intraperitoneum; MI, myocardial infarction; LA, left atrium; LAD, left anterior descending; LV, left ventricle; NE, norepinephrine; RA, right atrium; SHR, spontaneous hypertensive rats; ↔, no difference; ↓, reduced; ↑, increased.

**Table 5:** Effects of ivabradine on interstitial fibrosis in experimental heart failure.

Models	Dose and Duration of Ivabradine	Findings	Reference
Experimental HFpEF in mice	10 mg/kg/d (low) and 20 mg/kg/d (high) (p.o.) for 4 weeks	High dose: ↓ LV fibrotic area	[58]
Experimental HFrEF in mice	10 mg/kg/d and 20 mg/kg/d (p.o.) for 8 weeks	High dose: ↓ LV fibrotic area ↓ LV α-SMA protein ↓ LV CTGF protein ↓ LV Col-1 and Col-3 protein ↓ LV TGF-β1 protein ↓ LV TGFR2 protein ↓ LV pSmad2/3 protein Low dose: ↓ LV fibrotic area ↓ LV α-SMA protein ↓ LV CTGF protein ↓ LV Col-1 and Col-3 protein ↓ LV TGF-β1 protein ↓ LV TGFR2 protein ↓ LV pSmad2/3 protein	[58]
Ang II-induced primary ventricular fibroblast proliferation	3 and 10 µM	Both concentrations: ↓ fibroblast proliferation rate ↓ α-SMA protein ↓ CTGF protein ↓ Col-1 and Col-3 protein ↓ TGF-β1 protein ↓ TGFR2 protein ↓ pSmad2/3 protein	[58]
Cardiogenic-shock-induced cardiac remodeling in pigs	0.3 mg/kg (i.v. bolus)	↓ MMP-9 protein ↔ *EMMPRIN* mRNA ↓ EMMPRIN protein ↑ EMMPRIN+Cav3 colonization	[74]
Isoproterenol-induced HF in rats	10 mg/kg/d (p.o.) for 14 days	↓ serum MMP-9	[55]
Hyperthyroid cardiomyopathy in rats	10 mg/kg/d (p.o.) for 28 days	↔ cardiac fibrosis	[73]
Myocardial I/R-induced cardiac remodeling in pigs	0.3 mg/kg for 7 days	↓ MMP-9 protein	[71]
Diastolic-dysfunction-induced HF in diabetic mice	20 mg/kg/d in drinking water for 4 weeks	↓ α-SMA protein ↓ Collagen 1 protein ↓ Collagen 3 protein ↓ TIMP2 protein ↑ MMP2 protein	[56]
High-glucose-treated rat primary ventricular cardiac fibroblasts	10–40 µM	All concentrations: ↓ Collagen 1 protein ↓ Collagen 3 protein ↓ α-SMA protein ↓ TIMP2 protein ↓ MMP2 protein	[56]
Diabetic cardiomyopathy in mice	20 and 40 mg/kg/d in drinking for 12 weeks	Both doses: ↓ collagen	[106]
Transverse-aortic-constriction- induced cardiac hypertrophy in mice	10, 20, 40, and 80 mg/kg/d (i.g.) for 4 weeks	All doses: ↓ *Col 1* mRNA ↓ *Col 3* mRNA ↓ PI3K protein ↓ mTORC1 ↔ mTORC2 ↓ Akt ↓ p-Akt ↓ p-p70S6K1	[15]
Diabetic cardiomyopathy in mice	20 mg/kg/d (p.o.) for 12 weeks	↓ Col 1 protein ↓ Col 3 protein	[13]
SU5416+Hypoxia-induced cardiac remodeling in rats	10 mg/kg/d (p.o.) for 3 weeks	↓ RV collagen area ↓ RV collagen I/III protein ratio ↓ RVTGF-β1 protein ↓ RV pSMAD2/Smad2,3 protein ↓ RV pSMAD3/Smad2,3 protein ↓ RV CTGF protein	[72]
RV pressure-loaded-induced cardiac remodeling in rats	10 mg/kg/d (p.o.) for 3 weeks	↓ RV collagen area ↓ RV collagen I/III protein ratio ↓ RVTGF-β1 protein ↓ RV pSMAD2/Smad2,3 protein ↓ RV pSMAD3/Smad2,3 protein ↓ RV CTGF protein	[72]
Pulmonary-hypertension-induced cardiac remodeling in rats	10 mg/kg/d (p.o.) for 3 weeks	↓ RV collagen area ↓ RV collagen I/III protein ratio ↓ RVTGF-β1 protein ↓ RV pSMAD2/Smad2,3 protein ↓ RV pSMAD3/Smad2,3 protein ↓ RV CTGF protein	[72]
Experimental hypertension-induced cardiac remodeling in SHR	10 mg/kg/d in drinking water for 6 weeks	↓ LV collagen ↓ LV hydroxyproline	[53]
Experimental hypertension-induced cardiac remodeling in rats	10 mg/kg/d in drinking water for 4 weeks	↔ LV hydroxyproline	[62]
Isoproterenol-induced HF in rats	10 mg/kg/d (p.o.) for 6 weeks	↓ LV hydroxyproline ↓ LV collagen	[54]
LAD coronary-artery-ligated- induced cardiac remodeling in rats	6–8 mg/kg/d (i.p.) for 4 weeks	↓ LV collagen ↓ LV TGF-β protein ↔ LV VEGF-A protein ↔ LV bradykinin protein	[66]
Severe post-MI chronic HF in rats	10 mg/kg/d in drinking water for 3 months	↓ collagen volume fraction	[63]
Hypertension-induced HF in rats	10 mg/kg/d in drinking water for 10 weeks	↔ LV *Col 1a1* gene ↔ RA *Col 1a1* gene ↔ LA *Col 1a1* gene	[11]
Abdominal-aorta-constriction- induced chronic heart failure in rats	10 mg/kg/d (p.o.) for 12 weeks	↓ CTGF protein ↓ *TGF-β1* gene ↓ *COL-1* gene	[12]

Akt, protein kinase; Cav3, caveolin 3; Col 1, collagen 1; Col 3, collagen 3; CTGF, connective tissue growth factor; EMMPRIN, extracellular matrix metalloproteinase inducer; HFpEF, heart failure with preserved ejection fraction; HFrEF, heart failure with reduced ejection fraction; i.g., intragastric; i.p., intraperitoneum; i.v., intravenous; LV, left ventricle; MMP, matrix metalloproteinase; mTORC, mammalian target of rapamycin complex; p-Akt, phosphorylated protein kinase; p.o., per oral; PI3K, phosphatidylinositol 3-kinase; p-p70S6K1, phosphorylated protein S6 kinase beta-1; α-SMA, α-smooth muscle actin; SU5416, a tyrosine kinase inhibitor; TGF-β1, transforming growth factor β1; TGFR2, transforming growth factor receptor 2; pSmad (or pSMAD), phosphorylated small mothers against decapentaplegic; TIMP, tissue inhibitor of metalloproteinase; VEGF-A, vascular endothelial growth factor A; ↔, no difference; ↓, reduced; ↑, increased.

**Table 6:** Effects of ivabradine on apoptosis, autophagy, and biogenesis in animal studies.

Models	Dose and Duration of Ivabradine	Findings	Reference
LAD coronary-artery-ligated- induced cardiac remodeling in rats	10 mg/kg/d in drinking water for 90 days	↑ energy charge, ↑ creatine phosphate ↓ ADP, ↔ ATP, ↔ AMP	[14]
LAD coronary-artery-ligated- induced cardiac remodeling in rats	10 mg/kg/d (i.g.) for 7 days	↑ LC3II/I protein ↓ p62 protein ↑ beclin 1 protein ↑ ATG5 protein, ↑ ATG7 protein ↓ p-PI3K, ↓ p-AKT ↓ p-mTOR, ↓ p-p70S6K	[67]
Transverse-aortic-constriction- induced cardiac hypertrophy in mice	10, 20, 40, and 80 mg/kg/d (i.g.) for 4 weeks	All doses: ↓ cleaved caspase-3 protein ↑ caspase-3 protein ↓ PI3K protein ↓ Akt, ↓ p-Akt, ↓ p-p70S6K1	[15]
Diabetic cardiomyopathy in mice	20 and 40 mg/kg/d in drinking for 12 weeks	Both doses: ↓ TUNEL	[107]
Diabetic cardiomyopathy in mice	20 mg/kg/d (p.o.) for 12 weeks	↓ cleaved caspase-3 protein ↓ TUNEL	[13]

Akt, protein kinase; AMP, adenosine monophosphate; ADP, adenosine diphosphate; ATG, autophagy-related; ATP, adenosine triphosphate; i.g., intragastric; LAD, left anterior descending; LC3II/I, microtubule-associated protein light chain 3 II/I; mTOR, mammalian target of rapamycin; p-Akt, phosphorylated protein kinase; PI3K, phosphatidylinositol 3-kinase; p.o., per oral; p-p70S6K1, phosphorylated protein S6 kinase beta-1; TUNEL, terminal deoxynucleotidyl transferase dUTP nick-end labeling; ↔, no difference; ↓, reduced; ↑, increased.

**Table 7:** Effects of ivabradine on inflammation and oxidative stress in patients with heart failure and animal studies.

Subjects	Dose of Ivabradine	Findings	Reference
Patients with cardiomyopathy (n = 33)	5 mg b.i.d. for 3 months and 7.5 mg b.i.d. for 3 months	↓ TNFα, ↔ IL-6 ↔ hsCRP	[32]
Patients with cardiomyopathy (n = 33)	5 mg b.i.d. for 3 months and 7.5 mg b.i.d. for 3 months	↔ sST2, ↓ GDF-15 ↓ H-FABP, ↔ suPAR	[124]
Patients with HF (n = 10)	5 mg b.i.d. and titrated to 7.5 mg b.i.d. for 6 months	↓ IL-6, ↓ TNFα	[34]
Diabetic cardiomyopathy in mice	20 mg/kg/d (p.o.) for 12 weeks	↓ Heart *TNF-α* mRNA and protein ↓ Heart *IL-1β* mRNA and protein ↓ Heart *IL-6* mRNA and protein ↓ p-JNK, ↓ p-38	[13]
High-glucose-treated rat primary ventricular cardiac fibroblasts	10–40 µM	All concentrations: ↓ p-JNK protein ↓ p-p38 protein ↓ cell proliferation	[56]
Diastolic-dysfunction-induced HF in diabetic mice	20 mg/kg/d in drinking water for 4 weeks	↓ p-JNK protein ↓ p-p38 protein	[56]
LAD coronary-artery-ligated- induced cardiac remodeling in rats	10 mg/kg/d (i.g.) for 7 days	↓ TNFα ↓ IL-1β ↓ IL-6	[67]
Hypertension-induced cardiac remodeling in SHR	1 mg/kg/d (i.p.) for 14 days	↓ Number of inflammatory nuclei	[70]
Myocardial I/R-induced cardiac remodeling in pigs	0.3 mg/kg for 7 days	↑ de-expression of heart CyPA ↓ plasma CyPA protein ↑ cardiac CyPA protein ↔ heart *CyPA* mRNA ↑ CyPA-LG-EMMPRIN protein ↔ CyPA degradation	[71]
Hyperthyroid cardiomyopathy in rats	10 mg/kg/d (p.o.) for 28 days	↔ cardiac inflammation	[73]
High-glucose-induced apoptosis in cardiomyocytes	1–40 μM (pretreatment)	5–40 μM: ↓ p-IKKα/β protein ↔ IKKβ protein ↓ p-IκBα protein ↑ t-IκBα protein ↑ cyto NF-κB protein ↓ nuclear NF-κB protein	[107]
Diabetic cardiomyopathy in mice	20 and 40 mg/kg in drinking water for 12 weeks	Both doses: ↓ p-IKKα/β protein ↔ IKKβ protein ↓ p-IκBα protein ↑ t-IκBα protein ↑ cyto NF-κB protein ↓ nuclear NF-κB protein	[107]
Abdominal-aorta- constriction-induced chronic heart failure in rats	10 mg/kg/d (p.o.) for 12 weeks	↑ SOD protein	[12]

b.i.d., twice daily; cyto, cytoplasmic; CyPA, cyclophilin A; LG-EMMPRIN, low-glycosylated extracellular matrix metalloproteinase inducer; GDF-15, growth-differentiation factor-15; H-FABP, heart-type fatty acid binding protein; hsCRP, high-soluble C-reactive protein; i.g., intragastric; IL-1β, interleukin 1β; IL-6, interleukin 6; i.p., intraperitoneum; NF-κB, nuclear factor kappa-B; p-IκBα, phospho-nuclear factor of kappa light polypeptide gene enhancer in B-cells inhibitor α; t-IκBα, total inhibitor of nuclear factor kappa-B kinase subunit α/β; IκBα, nuclear factor of kappa light polypeptide gene enhancer in B-cells inhibitor α; p-IKKα/β, inhibitor of nuclear factor kappa-B kinase subunit α/β; p-JNK, phosphorylated c-Jun N-terminal kinase; p.o., per oral; p-p38, phosphorylated p38; SOD, superoxide dismutase; sST2, soluble suppression of tumorigenicity; suPAR, soluble urokinase plasminogen activator receptor; TNFα, tumor necrosis factor α; ↔, no difference; ↓, reduced; ↑, increased.

**Table 8:** Effects of ivabradine on cardiac structure and hypertrophy in patients with heart failure and animal studies.

Subjects	Dose of Ivabradine	Findings	Reference
Patients with chronic HF (n = 30)	5 mg b.i.d. for 4 months	↓ LVESD ↔ LVEDD	[36]
Acute HF patients with inflammatory rheumatic disease (n = 12)	2.5 mg/d b.i.d. titrated to 5 mg/d b.i.d. for 2 weeks	↓ Plasma NT-proBNP	[23]
Patients with HFpEF (n = 84) (EDIFY study)	Started at 5 mg b.i.d. and titrated to 7.5 mg b.i.d. or 2.5 mg b.i.d. for 8 months	↔ NT-proBNP ↔ LV mass index	[27]
Patients with systolic chronic HF (n = 98)	Started at 5 mg b.i.d. and titrated to 7.5 mg b.i.d. or 2.5 mg b.i.d. for 6 months	↓ Cystatin-C, ↓ CA-125 ↓ NT-proBNP	[29]
Patients with systolic HF (n = 43)	NA (3 months)	↔ PW, IVS, ↔ LA diameter	[44]
Patients with cardiomyopathy (n = 33)	5 mg b.i.d. for 3 months and 7.5 mg b.i.d. for 3 months	↔ LVEDD ↔ serum BNP	[32]
Hospitalized patients with acute decompensated systolic heart failure (n = 10)	Started at 5 mg b.i.d. and titrated to 7.5 mg b.i.d. or 2.5 mg b.i.d. until discharged	↓ NT-ProBNP	[33]
Patients with HF (n = 10)	5 mg b.i.d. and titrated to 7.5 mg b.i.d. for 6 months	↓ NT-ProBNP	[34]
Patients with chronic HF (n = 1873)	5 mg b.i.d. and titrated to 7.5 mg or 2.5 mg b.i.d. for 4 months	↓ BNP	[35]
Patients with chronic HF (n = 767) (RELIf-CHF study)	5 mg b.i.d. and titrated to 7.5 mg or 2.5 mg b.i.d. for 12 months	↓ BNP	[20]
Transverse-aortic-constriction-induced cardiac hypertrophy in mice	10, 20, 40, and 80 mg/kg/d (i.g.) for 4 weeks	All doses: ↓ IVSd, ↓ LVIDs, ↓ LVPWd ↓ LV mass, ↓ HW/BW 10 and 20 mg/kg/d: ↓ IVSs, ↓ LVIDd	[15]
Open chest with LV post-ischemia dysfunction in pigs	Bolus infusion of 0.5 mg/kg	↑ LVED dimension	[64]
Chronic ischemic heart failure in diabetic rats	10 mg/kg/d (i.p.) for 7 weeks	↓ LVDD, ↓ LVSD, ↓ BNP	[65]
LAD coronary-artery-ligated-induced cardiac remodeling in rats	10 mg/kg/d in drinking water for 90 days	↓ HW, ↓ ANP	[14]
LAD coronary-artery-ligated-induced cardiac remodeling in rats	6–8 mg/kg/d (i.p.) for 4 weeks	↑ LVW/BW ↑ LV coronary reserve ↓ LV arteriolar length	[66]
Hypertension-induced cardiac remodeling in SHR	1 mg/kg/d (i.p.) for 14 days	↔ LVW/BW ↓ cardiomyocyte diameter	[70]
Myocardial I/R-induced cardiac remodeling in pigs	0.3 mg/kg for 7 days	↓ heart necrosis	[71]
Pulmonary-hypertension-induced cardiac remodeling in rats	10 mg/kg/d (p.o.) for 3 weeks	↔ HW/BW ↓ cardiomyocyte diameter	[72]
RV pressure-loaded-induced cardiac remodeling in rats	10 mg/kg/d (p.o.) for 3 weeks	↔ HW/BW, ↓ RVEDD ↓ cardiomyocyte diameter	[72]
SU5416+Hypoxia-induced cardiac remodeling in rats	10 mg/kg/d (p.o.) for 3 weeks	↓ HW/BW, ↓ RVEDD ↓ cardiomyocyte diameter	[72]
Hypertension-induced HF in rats	10 mg/kg/d in drinking water for 10 weeks	↓ LV IVST, ↓ LVDD ↓ LVSD, ↓ LVSWS ↓ LV mass, ↓ LA dimension ↓ LV/BW, ↓ LA/BW ↓ LV *ANP* gene ↓ LA *ANP* gene ↔ RA *ANP* gene ↔ LV *β-MHC* gene	[11]
Hyperthyroid cardiomyopathy in rats	10 mg/kg/d (p.o.) for 28 days	↓ HW/BW ↔ cardiomyocyte width ↓ LVESD, ↔ LVEDD ↓ LA dimension, ↔ IVSd	[73]
Myocardial I/R-induced cardiac remodeling in rats	10 mg/kg/d (p.o.) for 28 days	↔ LVIDd, ↔ LVIDs	[57]
MI-induced cardiac remodeling in rats	10 mg/kg/d in drinking water for 8 weeks	↑ HW/BW, ↑ LV/BW ↑ posterior wall-end diastolic thickness	[61]
Severe post-MI chronic HF in rats	10 mg/kg/d in drinking water for 3 months	↓ LA dimension, ↓ VW/BW	[63]
Isoproterenol-induced HF in rats	10 mg/kg/d (p.o.) for 14 days	↓ serum NT-proBNP	[55]
Experimental HFpEF in mice	10 mg/kg/d (low) and 20 mg/kg/d (high) (p.o.) for 4 weeks	High dose: ↓ cardiomyocyte size ↓ HW/TL, ↓ IVSd, ↓ LVPWd	[58]
Experimental HFrEF in mice	10 mg/kg/d and 20 mg/kg/d (p.o.) for 8 weeks	High dose: ↓ cardiomyocyte size, ↓ HW/TL	[58]
Experimental hypertension-induced cardiac remodeling in rats	10 mg/kg/d in drinking water for 4 weeks	↔ VW/BW	[62]
Isoproterenol-induced HF in rats	10 mg/kg/d (p.o.) for 6 weeks	↓ LVW/BW, ↔ RVW/BW	[54]
Experimental hypertension-induced cardiac remodeling in SHR	10 mg/kg/d in drinking water for 6 weeks	↔ LVW/BW	[53]
Cardiogenic-shock-induced cardiac remodeling in pigs	0.3 mg/kg (i.v. bolus)	↓ c-Tn-1	[74]

ANP, atrial natriuretic peptide; b.i.d., twice daily; BNP, B-type natriuretic peptide; BW, body weight; c-Tn-1, cardiac troponin 1; CA-125, cancer antigen 125; HF, heart failure; HW, heart weight; i.g., intragastric; i.p., intraperitoneum; i.v., intravenous; IVS, intraventricular septum; IVSs, interventricular septal thickness at systole; IVSd, interventricular septal thickness at diastole; IVST, interventricular septal thickness; LA, left atrium; LAD, left anterior descending; LV, left ventricle; LVDD, left ventricular diastolic dimension; LVEDD, left ventricular end-diastolic diameter; LVESD, left ventricular end-systolic diameter; LVIDd, left ventricular internal diameter at diastole; LVIDs, left ventricular internal diameter at systole; LVPWd, left ventricular posterior wall thickness at diastole; LVSD, left ventricular systolic dimension; LVSWS, left ventricular end-systolic wall stress; LVW, left ventricular weight; VW, ventricle weight; β-MHC, β-myosin heavy chain; NT-proBNP, N-terminal proBNP; p.o., per oral; PW, posterior wall thickness; RA, right atrium; RVEDD, right ventricular end-diastolic diameter; RVW, right ventricular weight; SU5416, a tyrosine kinase inhibitor; TL, tibial length; ↔, no difference; ↓, reduced; ↑, increased.

## Data Availability

Not applicable.
